# Parental perceptions of informed consent in a study of tracheal intubations in neonatal intensive care

**DOI:** 10.3389/fped.2023.1324948

**Published:** 2024-01-08

**Authors:** Susanne Tippmann, Janine Schäfer, Christine Arnold, Julia Winter, Norbert W. Paul, Eva Mildenberger, André Kidszun

**Affiliations:** ^1^ Division of Neonatology, Department of Pediatrics, University Medical Center of the Johannes Gutenberg-University Mainz, Mainz, Germany; ^2^ Division of Neonatology, Department of Pediatrics, Inselspital, Bern University Hospital, University of Bern, Bern, Switzerland; ^3^ Institute for History, Philosophy, and Ethics of Medicine, Johannes Gutenberg-University Medical Center, Mainz, Germany

**Keywords:** informed consent, deferred consent, neonatology, parents, resuscitation, research ethics

## Abstract

**Background and objective:**

Obtaining informed consent in neonatal emergency research is challenging. The aim of this study was to assess parental perceptions of informed consent following participation in a clinical trial in neonatal emergency care.

**Methods:**

This was a supplementary analysis of a randomised controlled trial comparing video and direct laryngoscopy for neonatal endotracheal intubation in the delivery room and neonatal intensive care unit. After obtaining informed consent for the clinical trial, parents were asked to answer a series of self-administered questions about their perceptions of clinical trial participation and the consent process. Informed consent had been given either before birth, after birth but before inclusion in the trial, or after inclusion in the trial.

**Results:**

We received responses from 33 mothers and 27 fathers (*n* = 60) of the 63 preterm and term infants who participated in the study. Fifty-three (89.8%, *n* = 59) parents agreed that infants should participate in clinical trials, and 51 (85%, *n* = 60) parents agreed that parents should be asked for informed consent. Fifty-three (89.8%, *n* = 59) parents felt that their infant's participation in this particular trial would be beneficial. Fifty-two (86.7%, *n* = 60) parents felt that the informed consent process was satisfactory. One parent (100%, *n* = 1) approached before birth, 23 parents (82.1%, *n* = 28) approached after birth but before enrolment and 26 (83.9%, *n* = 31) parents approached after enrolment were satisfied with the timing of the consent process. Eight (13.3%, *n* = 60) parents felt some pressure to provide informed consent. Of these, two (25%) were approached before enrolment and six (75%) were approached after enrolment.

**Conclusion:**

Parents valued their infant's participation in an emergency neonatal clinical trial and considered it important to be asked for consent. In this study, it seemed less important whether consent was obtained before or after the intervention. Future studies may need to investigate which form of consent is most acceptable to parents for the individual study in question.

## 1 Introduction

Obtaining informed consent in neonatal emergency research is challenging. In general, when scientific studies are conducted with their children, parents should provide fully informed consent to participate before the study begins. However, it can be difficult to obtain ethically appropriate prenatal parental consent for studies involving neonates, especially those conducted shortly after birth. As a result it is difficult to recruit a sufficient number of cases, and participants are usually selected with some bias (1–4). For emergency research, deferred consent is considered an ethically justifiable approach that is increasingly being used (5). Deferred consent means that a patient is randomized to a study arm on the basis of a scientific protocol approved by the responsible Ethics Committee. Informed consent to continue participation in the study and/or to collect data is obtained only after randomization. For studies involving neonates, informed consent will be obtained from parents or legal guardians. Deferred consent appears to be acceptable to parents, especially for trials in emergency settings, and in general appears to be preferable to no consent (6). Although some studies have examined different types of consent, parents' perceptions of their infants' participation in clinical trials, including the types of consent used in trials, especially those involving neonatal emergency care, have not been adequately represented (5, 7). The aim of this study was to assess parental perceptions of informed consent following participation in a clinical trial in neonatal emergency care.

## 2 Methods

We conducted a supplementary analysis of a randomized controlled trial comparing video and direct laryngoscopy for neonatal intubation. In this randomized controlled trial, infants who required endotracheal intubation in the neonatal intensive care unit or the delivery room were randomly assigned to video vs. direct laryngoscopy to be used for the first intubation attempt. The results of this trial have been published previously (8).

To ensure that representative populations were included in the study and to improve scientific validity, parental consent for this study was obtained either before birth, after birth but before enrolment, or after enrolment. Parents were not contacted at the same time for other research projects. The informed consent procedure was approved by the local ethics committee (Rhineland-Palatinate Medical Association, ID: 2019-14405) in accordance with the underlying study protocol. Participating parents were required to provide written informed consent. Eligible parents were informed of study participation both verbally and in writing. The original German parent information sheet was carefully translated to English (ST and AK) and is included in Supplementary 1.

Parents were asked to answer a series of self-administered questions about their perceptions of clinical trial participation and the consent process. Questionnaires were given to parents during the informed consent process. Parents were instructed to complete and return the questionnaire after their infant participated in the study. Parents were not given a specific timeframe to return the questionnaires, but usually returned them within a few days of intubation. As this was a sub-study of an RCT, only parents of infants who participated in the study were eligible. The questionnaires were anonymous to assure participating parents that their responses were voluntary and confidential. A basic set of non-identifying demographic variables was included. Answers were given on 3- or 4-point Likert rating scales. Three-point Likert rating scales were bipolar (yes/no, better/worse) and offered a neutral selection (don't know, unsure). Four-point Likert rating scales were unipolar (fully agree, agree, do not agree, do not agree at all). The questionnaire was carefully translated into English by two authors (ST and AK) and is included in Supplementary 2.

The analysis and presentation of the results are descriptive. We have calculated absolute and relative frequencies or, depending on the scale level, the mean with standard deviation. For some questions, the responses are presented in a dichotomous way (agree/disagree).

## 3 Results

### 3.1 Study population

We received responses from 60 parents (33 mothers, 27 fathers) of the 63 preterm and term infants who participated in the study. Due to the anonymous and individual nature of the survey, it is unclear in how many cases only one or both parents in a family responded. The demographics of the participating parents are shown in Table 1.

**Table 1:** Demographics of the participating parents.

Characteristic	*N* = 60 parents
Female, *n* (%)	33 (55.0)
Age, mean (SD)	34.0 (4.7)
Marital status, *n* (%)	
Married	46 (76.7)
Cohabiting	14 (23.3)
Singleton	0 (0.0)
Siblings present, *n* (%)	27 (45.0)
Education level^a^, *n* (%)	
Did not complete secondary school	15 (25.9)
Completed secondary school	10 (17.2)
Completed tertiary education	33 (56.9)
Gestational age (weeks) of infant in the trial, mean (SD)	30.1 (4.9)

SD, standard deviation.

^a^ *n* = 58.

Of the participating parents, one (1.6%) provided informed consent before birth, 28 (46.7%) after birth but before enrolment, and 31 (51.7%) after birth and after enrolment. Parents of 13 infants were approached for consent but finally declined study participation. There is an unknown number of parents who were informed about the study but ultimately did not enrol their child because, for example, intubation was not performed.

### 3.2 Parental perception of participation in clinical trials

Fifty-three (89.8%, *n* = 59) parents agreed that infants should participate in clinical trials. None of the parents felt that infants who participate in clinical trials receive worse care than infants who do not participate. Twelve (20.0%, *n* = 60) parents felt that infants who participate in clinical trials generally receive better care. Forty-eight parents (80.0%, *n* = 60) were not sure if the care was better or worse. Fifty-six parents (93.3%, *n* = 60) felt well informed about the purpose of the trial. Fifty-three (89.8%, *n* = 59) parents felt that their infant's participation in this particular study of neonatal intubation would be beneficial.

### 3.3 Parental perception of the necessity of informed consent

Fifty-one (85%, *n* = 60) parents agreed that parents should be asked for their consent to participate in research studies involving their children. Despite being in favor of participating in the study, a minority of six (10%, *n* = 60) parents would have preferred not to be asked for consent.

### 3.4 Parental perception of prospective consent vs. deferred consent

The majority of parents were satisfied with both forms of informed consent, irrespective of whether it was prospective or deferred consent. Twenty-four of the 29 parents (82.8%) who were approached prospectively found the process satisfactory, and 26 of the 31 families (83.9%) who were approached retrospectively found the process satisfactory.

### 3.5 Parental perception of the timing of informed consent

When asked about the best time to discuss consent with parents for emergency neonatal clinical trials, 20 (33%, *n* = 60) parents said it was before birth, while 40 (67%, *n* = 60) parents said it was after birth. When asked the same question about non-emergency neonatal trials in general, 29 (48.3%, *n* = 60) parents said it was before birth, while 31 (51.7%, *n* = 60) parents said it was after birth.

Parents were unsure whether they would want to be contacted before birth if the chance of their infant receiving the intervention was very low. When asked whether all parents should still be contacted before birth about the possibility of participating in the study if only 1 in 60 new-born infants needed to be intubated after birth, 31 (52.5%, *n* = 59) agreed and 28 (47.5%, *n* = 59) disagreed.

### 3.6 Dissatisfaction with the way in which information was provided

Fifty-two (86.7%, *n* = 60) parents were satisfied with the way in which information was provided, while eight (13.3%, *n* = 60) parents indicated that they were not satisfied. Of these 8 parents, 4 (50%) were approached after birth and before enrolment, and 4 (50%) were approached after birth and after enrolment. All of these 8 were also dissatisfied with the modality of the consent process (prospective or retrospective consent).

Eight (13.3%, *n* = 60) parents felt some pressure to agree to participate in the study. Of these, two (25%) were approached after birth and before enrolment, and six (75%) were approached after birth and after enrolment. One (12.5%) of these parents was dissatisfied with the informed consent process. Two (25%) of them were also dissatisfied with the timing of the consent process.

The complete responses of the participating parents to all of the study questions are presented in Table 2. A visualization in the form of stacked bar graphs is provided in Supplementary 3.

**Table 2:** Complete responses of the participating parents.

	*n* (%)
Do you think children should participate in clinical trials?^a^	
Yes	53 (89.8)
No	2 (3.4)
Unsure	4 (6.8)
Do you think children who participate in trials are cared for better or worse?	
Better	12 (20.0)
Worse	0 (0.0)
Unsure	48 (80%)
Do you have a good understanding of what the goal of this study is?	
Yes	56 (93.3)
No	0 (0.0)
Unsure	4 (6.7)
Do you think your child's participation in this study is beneficial?^a^	
Yes	53 (89.8)
No	0 (0.0)
Unsure	6 (10.2)
I have been informed about my child's participation in the study in the following ways.	
Before birth	1 (1.7)
After birth and before enrolment in the study	28 (46.7)
After birth and after enrolment in the study	31 (51.7)
I am satisfied with the timing of the consent.	
Do not agree at all	4 (6.7)
Do not agree	6 (10.0)
Agree	39 (65.9)
Strongly agree	11 (18.3)
I am satisfied with the type of consent provided	
Do not agree at all	3 (5.0)
Do not agree	5 (8.3)
Agree	38 (63.3)
Strongly agree	14 (23.3)
Did you feel pressure to give your consent to participate in the study?	
Do not agree at all	33 (55.0)
Do not agree	19 (31.7)
Agree	5 (8.3)
Strongly agree	3 (5.0)
I would have preferred not to have been asked if my child could participate in the study.	
Do not agree at all	30 (50.0)
Do not agree	24 (40.0)
Agree	6 (10.0)
Strongly agree	0 (0.0)
On average, only one in 60 newborns needs intubation after birth. Nevertheless, should all parents be informed about possible study participation before birth?^a^	
Do not agree at all	7 (11.9)
Do not agree	21 (35.6)
Agree	23 (39.0)
Strongly agree	8 (13.6)
In general, should parents be asked for their consent to participate in research projects/studies that affect their children?	
Yes	51 (85.0)
No	4 (6.7)
Unsure	5 (8.3)
In general, when do you think is the best time to discuss studies involving their infants with parents?	
Before birth	29 (48.3)
Immediately after birth	3 (5.0)
After birth	28 (46.7)
In emergency situations, when do you think is the best time to discuss studies with parents involving their infants?	
Before birth	20 (33.3)
Immediately after birth	6 (10.0)
After birth	34 (56.7)

^a^ *n* = 59.

## 4 Discussion

We examined the perceptions of parents whose infants participated in a neonatal emergency trial of endotracheal intubation in which different consent approaches could be used. We found that parents valued their infant's participation in this trial and that parents wanted to be asked for consent.

A deferred consent model appears to be a feasible option with which most parents were satisfied. This is consistent with previous neonatal research, for example the findings of Sloss et al. but also with trials in pediatric and adult patients (2, 9, 10). In our results, the underlying trial investigated an acute intervention and randomly there was about the same level of consent before and after enrolment. Our findings are also consistent with current arguments by researchers, clinicians, and ethicists that a deferred consent approach can be implemented without violating either research ethics or the best interests of the child, while ensuring that very fragile or sick patients still benefit from valid research (4, 11). In particular, the results of our study may address some of the concerns raised by neonatal care providers regarding the appropriateness of the deferred consent approach to ensuring parental autonomy (1).

In a hypothetical study of cardiothoracic compressions, deferred consent would be significantly more acceptable than elective trial questions such as the best feeding strategies of preterm infants. Deferred informed consent was regarded to be more acceptable the higher the urgency of a likely intervention was. At the same time, parents may feel more pressure to give consent. In that same study of hypothetical research scenarios, 28% of parents would feel pressure to consent (6). This appears to be supported by the results of our study, which investigated an acute emergency intervention. While the majority of parents who gave deferred consent were satisfied with the process, a significant minority (17%) were dissatisfied in some way, but the details of why they were dissatisfied were not recorded in our study.

There are drawbacks to the deferred consent approach. In this study, we observed that some parents felt pressured to agree to participate in this study. This warrants further investigation. Further exploration of this issue could begin with monitoring and evaluating the acceptability of the consent process alongside active trials, not just immediately after consent is given, as in our analysis. For example, it has been shown that participation in a neonatal study, although generally experienced positive, can be a source of prolonged stress (12). This could be complemented by an analysis of the perceptions of those parents who refused to participate in specific trials.

Not only parents in the deferred consent group, but also some of those in the prospective consent group felt pressured to consent when asked about their infant's enrollment in the study. This issue also requires specific attention in future trials. However informed consent is obtained, but particularly in the case of deferred consent, researchers and clinicians should be aware that some parents may feel coerced into giving consent without articulating it. Strategies to reduce potential pressure need to be developed and studied.

Recent research has shown that many parents of infants enrolled in a neonatal hypoxic ischemic encephalopathy trial preferred a prospective, brief, two-step oral consent process to deferred consent (13). In this scenario, parents are briefly informed of the relevant aspects of the study prior to the intervention and are initially asked for verbal consent. Detailed information and written consent are provided only after that. While this approach may solve some of the consent issues for neonatal emergency research, it may still not be feasible for unexpected emergencies that require immediate intervention, such as studying cardiopulmonary resuscitation strategies. Thus, for emergency neonatal trials, there appears to be no one-size-fits-all approach to the consent process that is most acceptable to parents.

In general, antenatal consent appears to be the most acceptable form of consent. At the same time, it should be noted that if the consent procedure is carried out immediately before birth, this could make parents feel anxious, anxious to make decisions without adequate time for reflection and without the opportunity to obtain more adequate information. The ability to make a neutral decision could be influenced by threatening circumstances (6, 14). Since there is only one parent who was informed before birth and whose infant was enrolled in the study, we do not have any data to support or refute this statement. When we asked parents in general whether they would like to be asked to participate in the study, even if the probability of participation was very low, about half were in favor and half were against. When asked about the best time to discuss consent with parents for emergency neonatal clinical trials, majority was in favor of after birth.

The acceptability of the consent process is certainly multifactorial and includes the how and by whom parents are approached, the quality of the informational materials, the nature of the intervention being studied, its potential effects, and the severity of the infant's illness (15). Furthermore, it needs to be considered, that parents are usually morally distressed trying to receive the best possible care for their child but unsure, whether or not participation in a clinical trial serves this purpose best (16).

It seems imperative to reflect on the approach to obtaining informed consent not only with parent representatives in general, but especially with parents who have experience with or have been affected by the specific issue at hand. For neonatal intubation trials, the deferred consent approach may seem acceptable to parents for the following reasons: (1) a life-saving procedure has been performed, parents may feel grateful, (2) most infants do well despite the need for endotracheal intubation, and (3) the procedure does not appear to have significant long-term consequences. We, however, have no evidence to support these assumptions.

This analysis has strengths and limitations. It is one of the few studies to examine parental perceptions of informed consent alongside research on neonatal emergencies that simultaneously included parents who provided both prospective and deferred informed consent. However, the parents of 13 infants who refused to participate in the study provided no input beyond their refusal. The anonymized approach did not allow further investigation of associations of procedures or infant outcomes with parents' views of trial participation or the consent process. Language barriers may have introduced a selection bias, as some parents may not have responded to the questionnaire because their first language is not German. Ethnicity was not asked about either. The small sample size and single-center design of the study also limit generalizability.

Future studies should qualitatively explore the underlying motivations, views, emotions, and values of affected parents, without which a deep understanding of the observed behaviors and attitudes is hardly possible. This could be particularly helpful for those parents who refuse to participate in a clinical trial. The involvement of representative groups of affected parents in the design and conduct of future studies seems essential.

## 5 Conclusion

Our study adds to the evidence that parents value their infant's participation in neonatal clinical trials and consider it important to be asked for consent. In this study, it seemed less important whether consent was obtained before or after the intervention. Acceptability of the consent approach may depend on the type of research and the severity of outcomes. Future studies may need to investigate what form of consent is most acceptable to parents before, during, and after the trial.

## Data Availability

The original contributions presented in the study are included in the article/**Supplementary Material**, further inquiries can be directed to the corresponding author.
