# Supplementary material for: Parental perceptions of informed consent in a study of tracheal intubations in neonatal intensive care
Source: Front Pediatr. 2024 Jan 8;11:1324948. doi: 10.3389/fped.2023.1324948 (PMC10800449; doi:10.3389/fped.2023.1324948)
Supplement: Supplementary file 1 [file Table1.docx]

Supplement 1

**1 The original German parent information sheet**

*The original text (in German) has been carefully translated*

|  | Principal investigator and contact person:  Dr. med. André Kidszun, M.A.  Neonatal Intensive Care Unit  Langenbeckstr. 1, 55131 Mainz  Phone:+49 (0) 6131 17-5892  andre.kidszun@unimedizin-mainz.de |
| --- | --- |
|  |  |

**Information for parents and parents-to-be**

**for the study entitled:**

**Videolaryngoscopy for nasotracheal intubation of newborns**

Dear Parents,

Nowadays, medical care for newborns is at a very high level. Nevertheless, medical measures can be further improved. We are conducting a study to explore one of these measures in more detail and **would like to ask you if you would like to participate in this study with your child.**

There are situations in which newborns have a severe breathing disorder after birth or in the first weeks of life. Sometimes it is then necessary to medically support the newborn's breathing. This is done by inserting a breathing tube into the trachea (as in general anesthesia) and using a ventilator to support or completely take over the respiratory function. Since the breathing tube (also called an endotracheal tube) is inserted into the trachea through the nose, the procedure is called "nasotracheal intubation".

This information leaflet will be given to you if nasotracheal intubation is planned or very likely to be performed on your child. In this case, you may consent or decline to participate in the study prior to the procedure. In rare cases - when nasotracheal intubation has already had to be performed in an emergency - you will not receive this information leaflet until after the intubation has been performed. If the intubation has already been performed, you can only consent to or decline participation in the study afterwards.

**What is the goal of the study?**

In order to be able to insert the breathing tube into the trachea, the doctor must see the entrance to the trachea. This requires a special spatula - a so-called laryngoscope. The aim of our study is to investigate whether a new technique - a laryngoscope connected to a video camera - makes it easier to insert the breathing tube.

We also want to learn how you, as parents, evaluate participation in such a study, especially with regard to how consent is obtained.

**How does the study work?**

In order to be able to precisely investigate scientifically whether the laryngoscope with the video camera (videolaryngoscope) is better, two examination groups must be formed. One group of newborns will be intubated with the previously known technique (classical or direct laryngoscopy), the other group with the new technique (videolaryngoscopy). The assignment to the groups is done randomly - so you cannot influence this.

Assignment to one or the other group determines which method will be used for the first attempt at intubation. If this attempt does not result in a succesful intubation, the physician can change the method for further attempts.

The intubation procedure will be recorded with a video camera in order to study the effects of the two methods in more detail. In particular, this will be used to investigate whether video laryngoscopy is less uncomfortable for the newborn than direct laryngoscopy.

**What are the risks of participating in the study?**

The risks of study participation are considered to be enormously low. Both methods are used by doctors in everyday clinical practice and comply with the applicable medical regulations. Your child will not suffer any additional stress due to blood samples or similar.

**Who benefits from this study?**

The findings of this study first serve the science. However, they could help to determine the most favorable method of intubation for future newborns.

**How does the informed consent process work?**

As already outlined at the beginning, information and consent are usually obtained before a child is intubated. Thus, you as parents will be addressed if your child has been admitted to the neonatal intensive care unit after birth or if it is already likely before birth that your child will be admitted there.

However, sometimes it is unforeseen necessary to intubate a newborn, e.g., in an emergency. Since it is particularly important to also examine these children in the study, an exception is made in these cases with regard to the timing of the informed consent. In these emergency situations, the child will be included in the study and you, as parents, will be asked for your consent as soon as it is possible (less than 24 hours *later*). If you do not give consent, you will of course not suffer any disadvantage and all study-related data (written data, video recordings) of the child will be deleted immediately upon request.

**How much effort does study participation mean for the individual participant?**

In the event of intubation, participation in the study does not mean any additional effort for your child. Due to the nature of the study, only video recording will be performed as an additional measure. As parents, we would like to ask you about study participation and the consent procedure by means of a questionnaire. This will take about 10 - 15 minutes.

**Voluntary participation and early termination**

Participation in this study is voluntary. You can withdraw your participation at any time before or during the study without giving reasons. In this case, if desired, your information and statements as well as all collected data and records of your child will be deleted.

**How is your and your child's personal information protected?**

Personal data about you and your child (e.g. age and gender) will be collected as part of the study. All data collected are used to be able to assess the study results correctly. All information and statements you make in the course of the study will be treated with absolute confidentiality. This means that all study staff comply with data protection regulations. Your data will be processed in accordance with Art. 6 of the state General Data Protection Regulation (DS-GVO). All data collected will be processed on access-protected computers and then stored safely and securely for ten years at the Center for Pediatric and Adolescent Medicine at Mainz University Medical Center. To ensure that your personal data and your child's health data cannot be traced back to you or your child, they are encrypted. This procedure is called pseudonymization. The data will not be passed on to third parties. The publication of the study results is anonymous. You have the right to obtain information about your personal data at any time (including the provision of a copy free of charge) and to request restriction, transfer, correction or deletion of this data. Furthermore, you can object to the processing of your data (Art. 13-21 DS-GVO).The person responsible for data processing is the study director Dr. med. André Kidszun. In case of discrepancies in data processing, you have the right to complain to a supervisory authority. The State Data Protection Commissioner RLP responsible for this can be reached at [poststelle@datenschutz.rlp.de.](mailto:poststelle@datenschutz.rlp.de)

**Insurance**

As a precautionary measure, you are advised that insurance has not been taken out for damages not culpably caused that may occur in connection with the study. Insurance coverage exists only if the physician or another employee of the study site is accused of culpable misconduct. In favor of the study participant, there may be an easing of the burden of proof in certain cases (German Civil Code § 630h: on the burden of proof in the case of liability for errors in information or treatment).
