# Supplementary material for: Parental perceptions of informed consent in a study of tracheal intubations in neonatal intensive care
Source: Front Pediatr. 2024 Jan 8;11:1324948. doi: 10.3389/fped.2023.1324948 (PMC10800449; doi:10.3389/fped.2023.1324948)
Supplement: Supplementary file 2 [file Table2.docx]

***Supplement 2***

**1 The Questionaire**

*The original text (in German) has been carefully translated*

|  | Principal investigator and contact person:  Dr. med. André Kidszun, M.A.  Neonatal Intensive Care Unit  Langenbeckstr. 1, 55131 Mainz  Phone:+49 (0) 6131 17-5892  andre.kidszun@unimedizin-mainz.de |
| --- | --- |
|  |  |

**Questionnaire on participation and education regarding the study - Videolaryngoscopy for nasotracheal intubation of newborns.**

Please rate the following statements and mark one answer per question.

Do you think children should participate in clinical trials?

yes ☐

no ☐

unsure ☐

Do you think children who participate in trials are treated better or worse?

better ☐

worse ☐

unsure ☐

Do you have a good understanding of what the aim of this study is?

yes ☐

no ☐

unsure ☐

Do you think your child's participation in this study is beneficial?

yes ☐

no ☐

unsure ☐

I have been informed about my child's participation in the study in the following ways.

informed

before birth ☐

after birth and before enrolment in the study ☐

after birth and after enrolment in the study ☐

I am satisfied with the timing of the consent

Do not agree at all ☐

Do not agree ☐

Agree ☐

Strongly agree ☐

I am satisfied with the type of consent provided

Do not agree at all ☐

Do not agree ☐

Agree ☐

Agree

Strongly agree ☐

Did you feel pressure to give your consent to participate in the study?

Do not agree at all ☐

Do not agree ☐

Agree ☐

Agree

Strongly agree ☐

I would have preferred not to have been asked if my child could participate in the study

disagree at all ☐

disagree ☐

agree ☐

Strongly agree ☐

On average, only one in 60 newborns needs intubation after birth. Nevertheless, should all parents be informed about possible study participation before birth?

disagree at all ☐

disagree ☐

agree ☐

Strongly agree ☐

In general, should parents be asked for their consent to participate in research projects/studies that affect their children?

Yes ☐

No ☐

unsure ☐

In general, when do you think is the best time to discuss studies involving their

infants with parents?

before birth ☐

immediately after birth ☐

after birth ☐

In emergency situations, when do you think is the best time to discuss studies with parents involving their infants?

before birth ☐

immediately after birth ☐

after birth ☐

Please give us some more information about yourself:

Age ___________ years

Gender female ☐ male ☐ diverse ☐

Marital status single ☐ married ☐ cohabiting ☐

What is your highest level of education?

Regular school ☐ High school diploma ☐ college ☐

Do you have other children? Yes ☐ No ☐

Number: ________

Pregnancy When was your current child born?

___________ weeks + ___________ days

Thank you for helping us challenge and improve our research approaches!
