# Supplementary material for: Parental perceptions of informed consent in a study of tracheal intubations in neonatal intensive care
Source: Front Pediatr. 2024 Jan 8;11:1324948. doi: 10.3389/fped.2023.1324948 (PMC10800449; doi:10.3389/fped.2023.1324948)
Supplement: Supplementary file 3 [file Table3.docx]

***n=59**
